# Structural and functional analysis reveals that human OASL binds dsRNA to enhance RIG-I signaling

**DOI:** 10.1093/nar/gkv389

**Published:** 2015-04-29

**Authors:** Mikkel Søes Ibsen, Hans Henrik Gad, Line Lykke Andersen, Veit Hornung, Ilkka Julkunen, Saumendra N. Sarkar, Rune Hartmann

**Affiliations:** 1 Centre for Structural Biology, Department of Molecular Biology and Genetics, Aarhus University, 8000 Aarhus C, Denmark; 2 Institute of Molecular Medicine, University Hospital, University of Bonn, Bonn 53127, Germany; 3 Department of Virology, University of Turku, 20520 Turku, Finland; 4 Viral Infections Unit, National Institute for Health and Welfare, 00300 Helsinki, Finland; 5 Department of Microbiology and Molecular Genetics, University of Pittsburgh Cancer Institute, Pittsburgh, PA 15213, USA

## Abstract

The oligoadenylate synthetase (OAS) enzymes are cytoplasmic dsRNA sensors belonging to the antiviral innate immune system. Upon binding to viral dsRNA, the OAS enzymes synthesize 2′-5′ linked oligoadenylates (2-5As) that initiate an RNA decay pathway to impair viral replication. The human OAS-like (OASL) protein, however, does not harbor the catalytic activity required for synthesizing 2-5As and differs from the other human OAS family members by having two C-terminal ubiquitin-like domains. In spite of its lack of enzymatic activity, human OASL possesses antiviral activity. It was recently demonstrated that the ubiquitin-like domains of OASL could substitute for K63-linked poly-ubiquitin and interact with the CARDs of RIG-I and thereby enhance RIG-I signaling. However, the role of the OAS-like domain of OASL remains unclear. Here we present the crystal structure of the OAS-like domain, which shows a striking similarity with activated OAS1. Furthermore, the structure of the OAS-like domain shows that OASL has a dsRNA binding groove. We demonstrate that the OAS-like domain can bind dsRNA and that mutating key residues in the dsRNA binding site is detrimental to the RIG-I signaling enhancement. Hence, binding to dsRNA is an important feature of OASL that is required for enhancing RIG-I signaling.

## INTRODUCTION

Efficient elimination of viral infection relies on both detection of the virus by pattern recognition receptors (PRRs) and inhibition of viral replication by antiviral restriction factors (1). Retinoic acid-inducible gene-I (RIG-I) is a PRR that upon activation by primarily 5′-triphosphate RNA induces a signaling cascade leading to interferon (IFN) gene expression (2–4). RIG-I is a DExD/H box RNA helicase that consists of two N-terminal caspase activation and recruitment domains (CARDs), a helicase domain and a C-terminal domain (CTD) also known as the regulatory domain. In the absence of viral RNA, RIG-I adopts an autoinhibited conformation. Upon 5′-pppRNA binding to the CTD and/or dsRNA binding to the helicase domain, RIG-I undergoes a conformational change that exposes the CARDs (5–7). The exposed CARDs bind K63-linked poly-ubiquitin (pUb), which in most cases is a prerequisite for efficient activation of the signaling pathway after binding to virus-derived RNA (8). However, binding of certain long 5′-triphosphorylated dsRNAs to RIG-I can elicit ubiquitin-independent signaling (9). The pUb can be covalently linked to RIG-I by the E3-ubiquitin ligase TRIM25 (10). Nonetheless, covalent attachment of pUb is not a requirement for RIG-I signaling as RIG-I can initiate signaling in a complex with unanchored pUb (8). The RIG-I-pUb complex forms tetramers (11,12), which engage with the CARD of the transmembrane adaptor protein mitochondrial antiviral-signaling protein (MAVS) located in the outer mitochondrial membrane (13–16). This causes MAVS to form prion-like aggregates that propagate a signal leading to activation of the transcription factors IRF3/IRF7 and thus to the activation of IFN gene expression (17,18).

**Figure 1.**
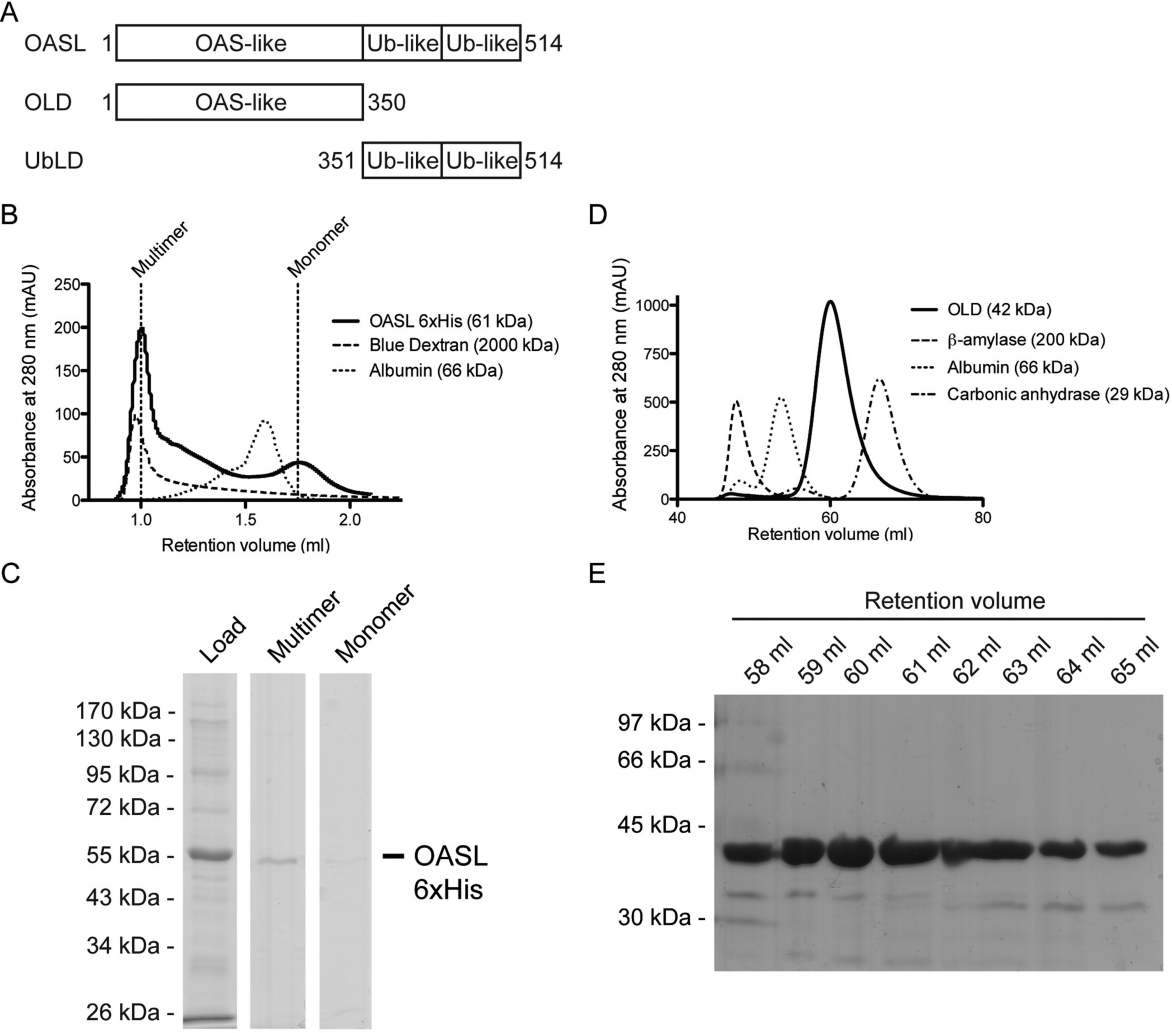
Full-length OASL forms soluble multimers while the OLD is a monomer. (**A**) Schematic representation of the domain organization of full-length OASL, the OLD and the UbLD. (**B**) Chromatograms of Superdex 200 PC3.2/30 analytical size exclusion chromatography of OASL, Blue Dextran and albumin in solid, dashed and dotted lines, respectively. ‘Multimer’ and ‘monomer’ indicate OASL elution peaks for multimeric and monomeric OASL, respectively. (**C**) A Coomassie stained 8% sodium dodecylsulphate-polyacrylamide gel electrophoresis (SDS-PAGE) analysis of the following: load: OASL loaded on the size exclusion chromatography column. Multimer: The OASL eluted in the void volume at ‘multimer’ as shown in (B). Monomer: Monomeric OASL eluted at ‘monomer’ as shown in (B). (**D**) Chromatograms of Superdex 75 HiLoad 16/60 size exclusion chromatography of OLD, β-amylase, albumin and carbonic anhydrase in solid, dashed, dotted and interchanging lines, respectively. (**E**) A Coomassie stained 10% SDS-PAGE analysis of retention volumes from the size exclusion chromatography shown in (D).

The 2′-5′ oligoadenylate synthetases (OASs) are a family of IFN- and virus-induced antiviral restriction factors (19–21) that provide protection against a wide spectrum of RNA and DNA viruses (for a review see (22)). The OAS family belongs to a nucleotidyltransferase (NTase) superfamily (23) and when activated by dsRNA the OAS enzymes synthesize 2′-5′ phosphodiester linked oligoadenylates (2-5As) with the general formula p_3_A(2′p5′A)*_n_* where *n* ≥ 1 (24,25). The OAS enzymes share structural homology and the ability to make 2′ specific phosphodiester bonds with the recently discovered PRR for cytosolic dsDNA, cyclic GMP-AMP synthase (cGAS) (26–31). In humans, the OAS family consists of four members: OAS1, OAS2, OAS3 and OASL (32,33). The active site of all polymerases (to which family the OAS enzymes belong) harbors a catalytic triad of three carboxyl acids (aspartic acid or glutamic acid), which coordinate the two magnesium ions required for catalysis (34). OAS1 binds dsRNA in a groove of positively charged residues (35,36). Upon binding to dsRNA, OAS1 undergoes a conformational change that repositions the catalytic triad to synthesize 2-5As (36,37). The 2-5As are second messengers that bind to and activate a latent endoribonuclease, RNase L (38,39), that subsequently restricts viral growth by degrading cellular and viral RNA (40). OAS1, -2 and -3 have 2′-5′ OAS activity (41). OASL, on the other hand, is devoid of enzymatic activity in part due to an incomplete catalytic triad in which the third carboxyl acid in position 152 has been substituted by a threonine (42,43). The 350 N-terminal residues of OASL have 39% amino acid identity with OAS1 and are known as the OAS-like domain (OLD; Figure 1A). Uniquely among the human OAS family members, OASL has two consecutive CTDs with homology to ubiquitin, which are therefore called ubiquitin-like domains (UbLDs; Figure 1A) (42). In spite of its missing enzymatic activity, OASL remains antiviral against a number of viruses and the antiviral activity of OASL requires the UbLD (44–48). Recently it was discovered that the UbLD of OASL could substitute for K63-linked pUb and interact with the CARDs of RIG-I (44). OASL thereby enhances RIG-I signaling leading to an amplified antiviral response. The findings describe a functional role of the UbLD, however, the role of the OLD has remained puzzling. In the present work, we describe the crystal structure of the OLD and address the functional roles of various structural elements of OASL in regard to RIG-I signaling enhancement.

## MATERIALS AND METHODS

### IFN-β promoter luciferase reporter assay

HEK293T cells, in which the OASL gene was knocked out by TALEN (OASL^neg^) as previously described (44), or HeLa cells, were seeded onto a 12-well plate (3×10^5^ cells/well) and cultured in Dulbecco's modified Eagle's medium (DMEM; Sigma-Aldrich) supplemented with 10% (v/v) fetal bovine serum (FBS; Sigma-Aldrich), 100 U/ml penicillin (Sigma-Aldrich) and 100 μg/ml streptomycin (Sigma-Aldrich) at 37°C in 5% CO_2_ for 24 h. The HEK293T OASL^neg^ cells in each well were transiently transfected with 1 μg of a reporter plasmid carrying a firefly luciferase gene downstream of an IFN-β promoter and 1 μg of a pcDNA3.1 OASL expression construct or the empty vector. The transfection reagent polyethylenimine (PEI; Polyplus transfection) was used in a 1:3 DNA:PEI ratio meaning that altogether 2 μg of plasmid DNA and 6 μg of PEI was used for each transfection. DMEM without FBS or antibiotics was added to the DNA and PEI up to a volume of 100 μl, respectively, and the DNA and PEI were mixed to give a total volume of 200 μl. The DNA–PEI mixture was incubated for 15 min at room temperature before gently being applied to the cells in one well. HeLa cells were transfected in the same manner as the HEK293T OASL^neg^ cells except that the HeLa cells were transfected with 320 ng IFN-β promoter firefly luciferase plasmid, 10 ng β-actin promoter *Renilla* luciferase plasmid and 670 ng of an empty vector or plasmids expressing different versions of OASL. 3 μg of PEI (Polyplus transfection) was used. After transfection, the HEK293T OASL^neg^ and HeLa cells were incubated for 24 h at 37°C before infection with 20 hemagglutination units (HAU) of murine Sendai virus (SeV; Cantell strain; National Institute of Health and Welfare, Helsinki, Finland) per well or left uninfected for 16 h. After infection, the cells were lysed with a passive lysis buffer (Promega). The lysates of the HEK293T OASL^neg^ were mixed with the luciferase substrate (Promega). To measure the firefly luciferase activity, relative light units (RLU) were measured with an illuminometer. The Dual-Luciferase® Reporter Assay System (Promega) was used to measure the firefly and *Renilla* luciferase activities in the lysates from the HeLa cells. Student's unpaired two-tailed t-test was used to assess statistical differences. Non-significant (Ns): *P*-value > 0.05; *: *P*-value ≤ 0.05; **: *P*-value ≤ 0.01; ***: *P*-value ≤ 0.001.

### Analytical size exclusion chromatography

The analytical size exclusion chromatography of OASL was carried out on a Superdex 200 PC3.2/30 column (GE Life Sciences). The column was calibrated with Blue Dextran (2000 kDa) and albumin (66 kDa) in phosphate buffered saline (PBS). The marker proteins were dissolved in PBS with 5% (v/v) glycerol. OASL was run in 50 mM Tris-HCl pH 9, 500 mM NaCl, 10% (v/v) glycerol, 1.5 mM MgCl_2_, 1 M urea and 1 mM β-mercaptoethanol (BME).

### Protein expression and purification

Recombinant full-length OASL was purified as previously described (44). OLD comprising the residues spanning 1–350 of OASL with a C-terminal 6xHis-tag was cloned into pET15b. *E. coli* BL21(DE3) (Novagen) was transformed with the construct and was grown at 37°C to an OD_600_ of 0.5–0.7 in lysogeny broth (LB) medium with 100 μg/ml ampicillin. The *E. coli* was cold-shocked for 20 min at 4°C before inducing protein expression with 0.5 mM isopropyl β-D-1-thiogalactopyranoside. OLD was expressed at 18°C over night (ON). The cells were harvested by centrifugation, washed in ice-cold PBS, resuspended in 10 ml per l culture of 50 mM NaH_2_PO_4_ pH 6.8, 300 mM NaCl, 20 mM imidazol, 10% (v/v) glycerol, 0.1% (v/v) NP-40, 5 mM BME and protease inhibitor cocktail (Sigma-Aldrich) and lysed by sonication. The lysate was cleared at 18 000 g at 4°C for 1 h. 1 ml of Ni-NTA-agarose (Qiagen) slurry per liter of culture was added to the supernatant for 1 h at 4°C with gentle shaking. The beads were cast into a column and washed with 10× column volume (CV) 50 mM NaH_2_PO_4_ pH 8, 500 mM NaCl, 20 mM imidazol, 10% (v/v) glycerol and 5 mM BME. Ni-NTA-agarose bound proteins were eluted with 10× CV 500 mM NaCl, 50 mM NaH_2_PO_4_ pH 6.8, 250 mM imidazol, 10% glycerol and 5 mM BME. The eluate was diluted 1:3 with buffer A (50 mM DL-malic acid pH 5.9, 50 mM NaCl, 2 mM BME and 10% (v/v) glycerol) and loaded onto a 5 ml HiTrap Heparin column (GE Life Sciences) with an ÄKTA prime (GE Life Sciences). The column was washed with buffer A and bound proteins were eluted in a linear gradient of buffer B (50 mM DL-malic acid pH 5.9, 1 M NaCl, 2 mM BME and 10% (v/v) glycerol) on an ÄKTA purifier (GE Life Sciences). Fractions containing OLD were pooled and concentrated to a volume of 1 ml using a Vivaspin 6 with a molecular weight cut-off (MWCO) of 10 000 (Sartorius Stedim Biotech) and further purified by size exclusion chromatography in 100 mM DL-malic acid pH 5.9, 200 Na_2_SO_4_, 300 mM NaCl, 2 mM BME and 5% (v/v) glycerol on a HiLoad 16/60 Superdex 75 (GE Life Sciences). Fractions containing OLD were pooled and concentrated to 5 mg/ml using a Vivaspin 6 with a MWCO of 10 000 (Sartorius Stedim Biotech). OLD K66E was purified in a similar fashion but without the affinity chromatography step involving the HiTrap Heparin column. Porcine OAS1 was purified as previously described (49).

### OLD crystallization and data collection

OLD with a C-terminal 6xHis-tag was crystallized by sitting-drop vapor diffusion. Initial nanoliter crystallization screening was performed using a Mosquito Crystal robot (TTP Labtech). Crystallization conditions were screened by mixing 5 mg/ml OLD in a 96-well screening plate with the Index screen (Hampton), Structure screen (Molecular Dimensions), JCSG+ screen (Molecular Dimensions), MIDAS screen (Molecular Dimensions) and an in-house screen at both 18°C and 4°C. Several hits were obtained and to optimize crystallization, grid screens were set up with varying pH, salt concentrations and precipitant concentrations around the initial crystal conditions. The best diffracting crystals were obtained by mixing 1 μl 5 mg/ml OLD with 1 μl 100 mM Tris pH 7.5, 200 mM LiSO_4_ and 20% (w/v) polyethylene glycol (PEG) 3350. The reservoir volume was 1 ml and the crystallization was carried out in a 24-well sitting drop crystallization plate (Hampton). Crystal needles appeared after 12 h at 4°C. The crystals were cryo-protected in mother liquor with 15% (v/v) ethylene glycol and flash frozen in liquid nitrogen. Data collection was performed at the I911-3 beamline at MAX-lab, Lund, Sweden, at a wavelength of 0.97907 Å. A data set of 180 frames with a rotation of 1° was collected using a Mar Mosaic 225 detector at a distance of 140 mm.

### OLD structure determination, model building and refinement

The diffraction data were indexed and scaled using the XDS software package (50). The data were processed in the space group P1 2_1_ 1 with unit cell parameters a = 54.8 Å, b = 57.62 Å, c = 64.79 Å and α = 90°, β = 99.44°, γ = 90°. Molecular replacement was used to determine the structure. One molecule of the inactive porcine OAS1 structure (PDB: 1PX5) was used as the search model in Phaser (51). An initial model of OLD was built using AutoBuild from the Phenix software package (52). Missing residues were built manually in Coot (53) and the model was optimized by iterative cycles of manual model building and refinement using Refine from the Phenix software package (52). Structural comparison by superposition was performed using secondary-structure matching (SSM) (54) or least squares (LSQ) in Coot. Surface charge representations were generated with PDB2PQR (55,56) and Adaptive Poisson-Boltzmann Solver (APBS) (57). All crystal structure presentations were generated with PyMOL (The PyMOL Molecular Graphics System, Version 1.5.0.4 Schrödinger, LLC). The alignment was performed in Clustal (58) and colored in JalView (59). The Dali server (60) was used to estimate root-mean-square deviation (RMSD) and Z-score between the crystal structures of OLD and OAS1.

### OAS immunoprecipitation

Generation of stable transfected cell lines using the Flp-In^TM^ T-Rex^TM^ HEK293 system was performed as described (61). Two 150-mm dishes were seeded with Flp-In^TM^ T-REx^TM^ HEK293 cells (Life Technologies) stable transfected with 3xC-terminal FLAG-tagged wild type OASL, OLD, hOAS1 or non-transfected, respectively. The cells were grown to 90% confluence with an estimated 20×10^6^ cells per plate. Protein expression was induced by the addition of 1 μg/ml tetracycline for 24 h. The cells were scrapped off the dishes using a rubber policeman and washed twice in 10 ml ice-cold PBS. The cells were lysed in 4 ml of 50 mM HEPES pH 7.5, 10 mM MgCl_2_, 150 mM NaCl, 0.5% (v/v) NP-40, 10% (v/v) glycerol, 25 mM NaF, 1 mM NaVO4 and protease inhibitor cocktail (Sigma-Aldrich). The lysate was cleared by centrifugation at 20 000 g for 30 min at 4°C. The supernatant was further cleared by filtration through a 45 μm nylon filter. To avoid unspecific binding to the beads and thus false positive interactions, the lysate was pre-cleared with 20 μl rabbit IgG agarose (Sigma-Aldrich) slurry used as decoy beads for 1 h at 4°C with gentle rocking. The decoy beads were removed by centrifugation at 50 g and 20 μl anti-FLAG M2 magnetic beads (Sigma-Aldrich) slurry was added to the supernatant ON at 4°C with gentle rocking. The beads were washed with 3×1 ml 50 mM HEPES pH 7.5, 10 mM MgCl_2_, 150 mM NaCl and 10% (v/v) glycerol. The beads with immobilized OAS proteins were used in the OAS activity assay.

### OAS activity assay

The catalytic activity of the different OAS proteins were assayed in 20 mM HEPES pH 7.5, 10 mM MgCl_2_, 10 mM MnCl_2_, 0.2 mM DTT, 5% (v/v) glycerol, 0.1 mg/ml bovine serum albumin, 0.2 mM EDTA, 0.5 mM ATP, 0.5 mM GTP, 0.5 mM TTP, 0.5 mM UTP and 100 μg/ml poly(I:C) (synthetic dsRNA analog; GE Life Sciences) or 100 μg/ml salmon sperm dsDNA (Invitrogen). 1 μg of protein purified from *E. coli* or insect cells was used and incubated at 37°C for 2 h. Alternatively, 5 μl anti-FLAG M2 magnetic beads with immunoprecipitated (IP) protein expressed in Flp-In T-Rex HEK293 cells immobilized on the beads were used and incubated at 37°C for 5 h. The reaction was heat-inactivated at 95°C for 5 min. 20 U/μl alkaline phosphatase was added to dephosphorylate free nucleotides for 2 h at 37°C. The reaction was heat-inactivated at 95°C for 5 min. Reaction products were bound to a 1 ml Resource Q column (GE Life Sciences) in 20 mM Tris-HCl pH 7.5 using an ÄKTA prime (GE Life Sciences). Bound products were eluted in a linear gradient ranging from 0 to 700 mM NaCl in 20 mM Tris-HCl pH 7.5 using an ÄKTA purifier (GE Life Sciences).

### Immunoblotting

Proteins were separated on 8% SDS-PAGE or native PAGE. The proteins were then transferred to a polyvinylidene flouride (PVDF) membrane (Applichem) and blocked with 5% milk in Tris-buffered saline with 0.05% Tween-20 (TTBS) at room temperature for 1 h. The membrane was washed 3×5 min in TTBS and blotted with mouse anti-V5 (Invitrogen, catalog# R960-25), rabbit anti-GAPDH (Santa Cruz Biotechnology, catalog# sc-25778), mouse anti-His_4_ (Qiagen, catalog# 34670) or mouse monoclonal anti-FLAG (Sigma-Aldrich, catalog# F3165) at 4°C ON. The membrane was washed 3×5 min in 1% milk in TTBS. The bound primary antibodies were detected using a donkey anti-mouse horseradish peroxidase (HRP)-conjugated antibody (Jackson ImmunoResearch, catalog# 715-036-150) or a goat anti-rabbit HRP-conjugated antibody (Pierce, catalog# 31461) in 1% milk TTBS at room temperature for 1 h. After secondary blotting the membrane was washed 4×5 min in TTBS before visualizing the antibody bound proteins on Kodak film with SuperSignal West Dura (Thermo Scientific).

### OLD native PAGE electrophoretic mobility shift assay and RNA retention assays

Native PAGE electrophoretic mobility shift assay (EMSA) was performed to investigate the binding of dsRNA to OLD. All binding experiments were performed in 100 mM DL-malic acid pH 5.9, 200 Na_2_SO_4_, 300 mM NaCl, 1.5 mM MgCl_2_, 2 mM BME and 5% (v/v) glycerol in a total volume of 10 μl. OLD was kept constant at 2.4 μM (1 μg) and mixed with 0 μM, 0.13 μM, 0.27 μM, 0.54 μM, 1.1 μM, 2.2 μM, 4.3 μM or 8.6 μM (1 μg) 18 bp dsRNA. The negative control was performed with 8.6 μM 18 bp dsRNA and no OLD. The sequence of the 18 bp dsRNA is sense: 5′-GGCUUUUGACCUUUAUGC-3′ and antisense: 5′-GCAUAAAGGUCAAAAGCC-3′ (Sigma-Aldrich) (36). The 18 bp dsRNA was obtained by annealing the single-stranded RNAs. For the comparison of gel migration elicited by wild-type OLD or K66E OLD in complex with 18 bp dsRNA, 2.4 μM OLD was mixed with 8.6 μM 18 bp dsRNA and incubated at 37°C for 15 min to allow for complex formation. Hi-Density TBE sample buffer (Invitrogen) was added to a final concentration of 0.5×. The EMSA was performed on 6% DNA Retardation Gels (Invitrogen) at 100V using 0.5× Tris-borate-EDTA (TBE) as running buffer. The proteins were visualized by silver stain or immunoblotting with an anti-His_4_ antibody.

For the dsRNA retention experiments, the concentration of either 18 bp dsRNA or poly(I:C)-LMW (InvivoGen) was kept constant at 10 μM or 0.2 mg/ml, respectively, and incubated with 0, 7, 14, 28 or 56 μM OLD wt or K66E. The mixtures were incubated at 37°C for 15 min to allow for complex formation. Hi-Density TBE sample buffer (Invitrogen) was added to a final concentration of 0.5×. The 18 nt dsRNA was separated on a 12% agarose native gel at 10W for 80 min at 4°C using 0.5× TBE as running buffer. The poly(I:C)-LWM was separated on a 6% DNA Retardation Gel (Invitrogen) at 100V using 0.5× TBE as running buffer. The nucleic acids were visualized by staining with ethidium bromide. The experiments were performed at least three times.

## RESULTS

### Full-length OASL forms multimers

We expressed full-length OASL (61 kDa) using the baculovirus system and purified it from insect cells. During the purification we observed a limited solubility of OASL, however, a non-denaturing concentration of urea (2 M) was able to keep OASL soluble. To assess the quality of the purified OASL, analytical size exclusion chromatography was performed. The majority of the purified OASL eluted in the void volume indicating that OASL predominantly forms multimers in solution while only a minor fraction exists on a monomeric form (Figure 1B and C). To analyze whether the multimerization of OASL is an artifact arising from the heterologous expression, we performed size exclusion chromatography on lysates from Flp-In T-Rex HEK293 cells stable transfected with OASL. Like previously, the majority of OASL eluted as a multimer while a minor part eluted with an apparent mass of approximately 150 kDa (Supplementary Figure S1A and B). The limited solubility of full-length OASL prompted us to utilize another purification strategy. We expressed the OLD (42 kDa) in *E. coli* and purified it to ∼95% homogeneity by immobilized metal affinity chromatography, affinity chromatography and size exclusion chromatography. The purified OLD eluted as a monomer during size exclusion chromatography (Figure 1D and E).

### The structure of the OLD is remarkably similar to the activated form of OAS1

**Figure 2.**
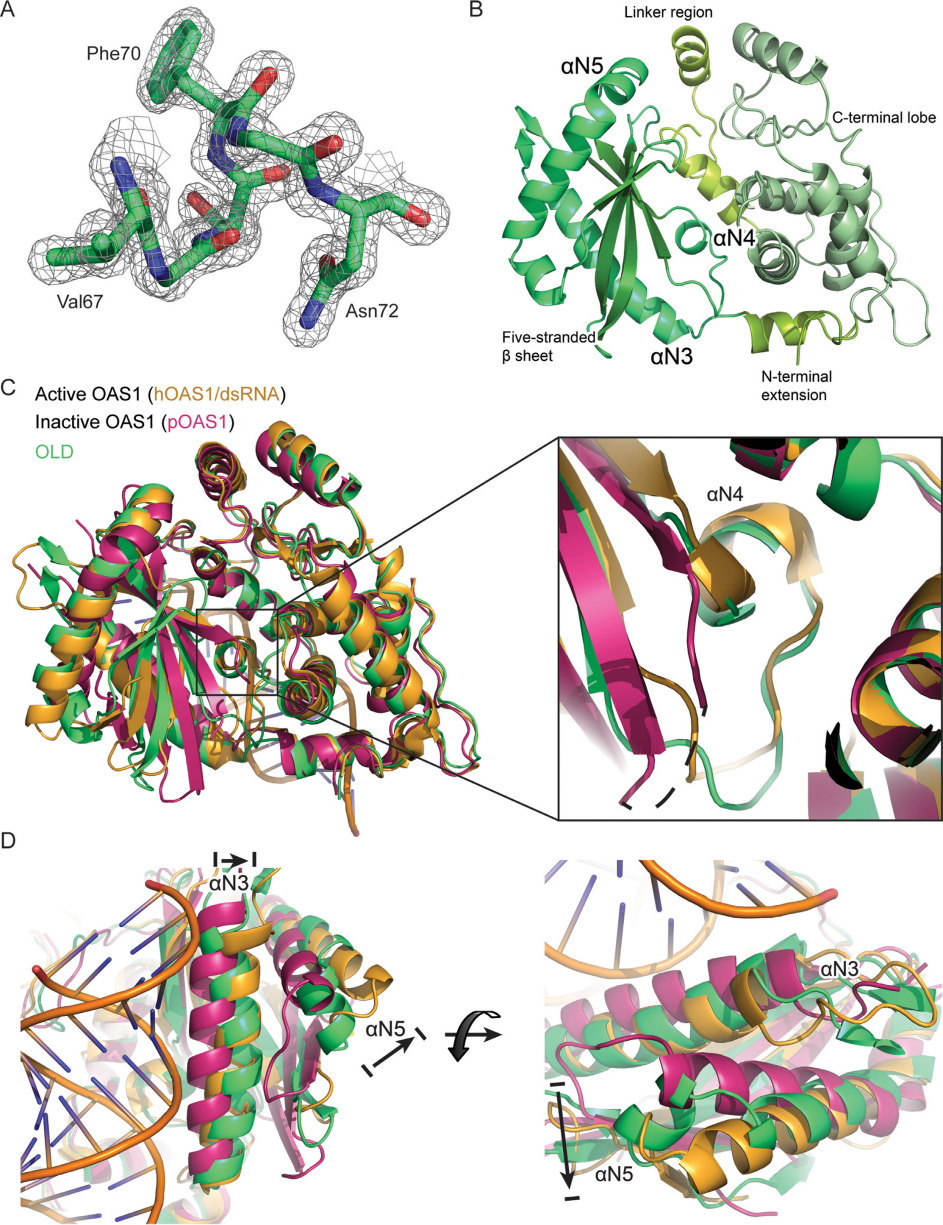
The structure of the OLD is remarkably similar to the activated form of OAS1. (**A**) Model building in the electron density contoured at 2σ. The displayed model and electron density include the αN4 helix. The side chains of Val67, Phe70 and Asn72 are indicated. (**B**) The crystal structure of OLD. The N-terminal extension and the linker regions that connect the N- and C-terminal lobes are indicated and colored yellow–green. The αN3, αN4 and αN5 helices are indicated and colored green. The five-stranded antiparallel β-sheet is indicated and colored dark green. The C-terminal lobe is indicated and colored light green. (**C**) Superposition of the OLD (green, PDB: 4XQ7) on active OAS1 (human OAS1 in complex with dsRNA, yellow, PDB: 4IG8) and inactive OAS1 (porcine OAS1, magenta, PDB: 1PX5). A zoom-in on the αN4 present in the active OAS1 and OLD but not in the inactive OAS1. The dashed line in the inactive OAS1 indicates the unstructured residues forming the αN4. (**D**) Superposition as in (C) showing the position of the αN3 and αN5 helices. The arrows indicate the RNA-induced rearrangement of αN3 and αN5 in OAS1.

**Figure 3.**
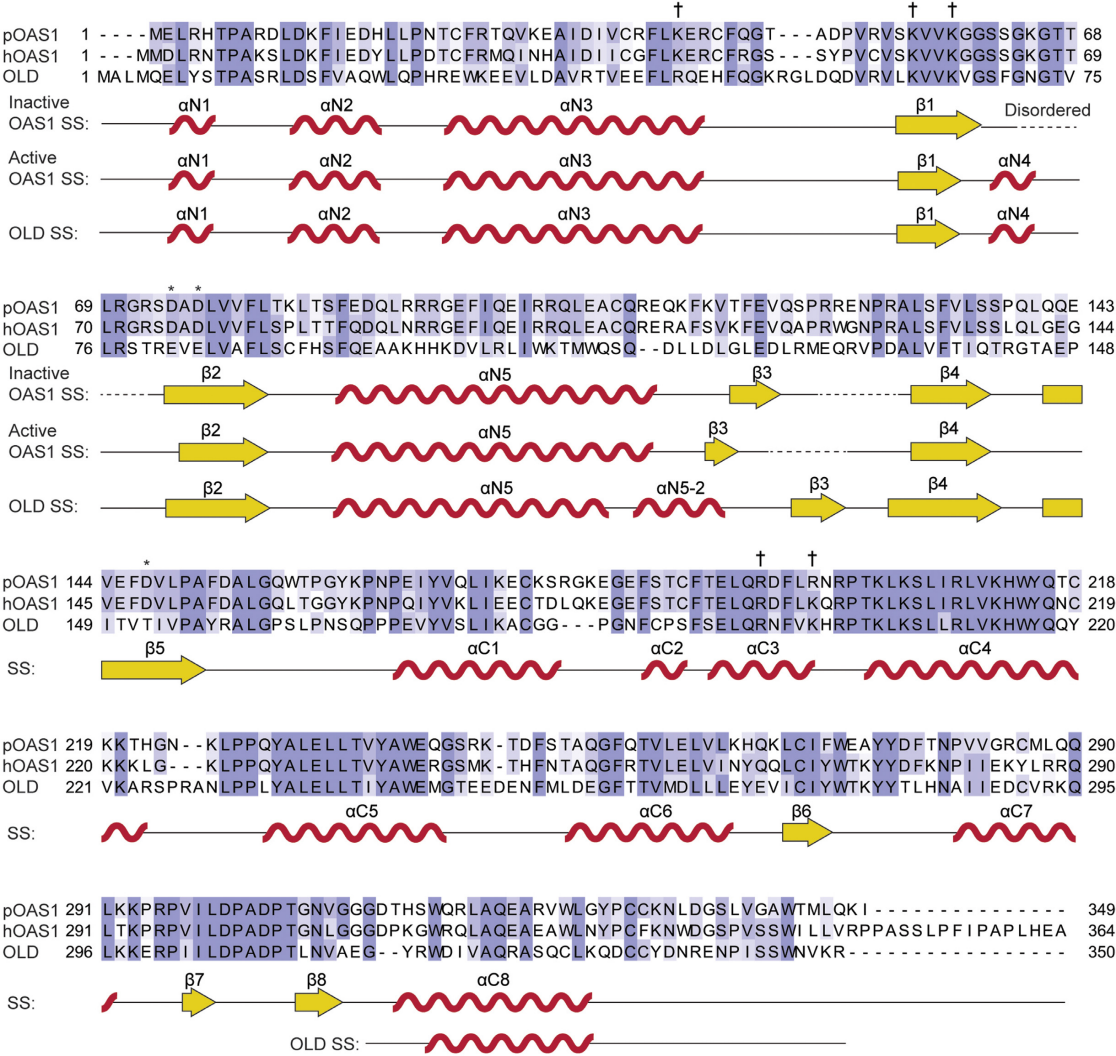
Shared and unique secondary structure features of OLD (PDB: 4XQ7) and active and inactive OAS1. The secondary structure is presented under the alignment and displays the shared secondary structure unless otherwise indicated. The structure of pOAS1 (PDB: 1PX5) was determined in the apo-form and thus represents the inactive OAS1 conformation. The structure of hOAS1 (PDB: 4IG8) was determined in complex with dsRNA and thus represents the active OAS1 conformation. α-helices are depicted as red waves and labeled according to position in the N-terminal (N) or C-terminal (C) lobe. β-sheets are depicted as yellow arrows and labeled according to overall position. Asterisks mark the catalytic triad of OAS1. Daggers mark residues important for binding to dsRNA. Dotted lines indicate disordered regions. SS: secondary structure.

**Figure 4.**
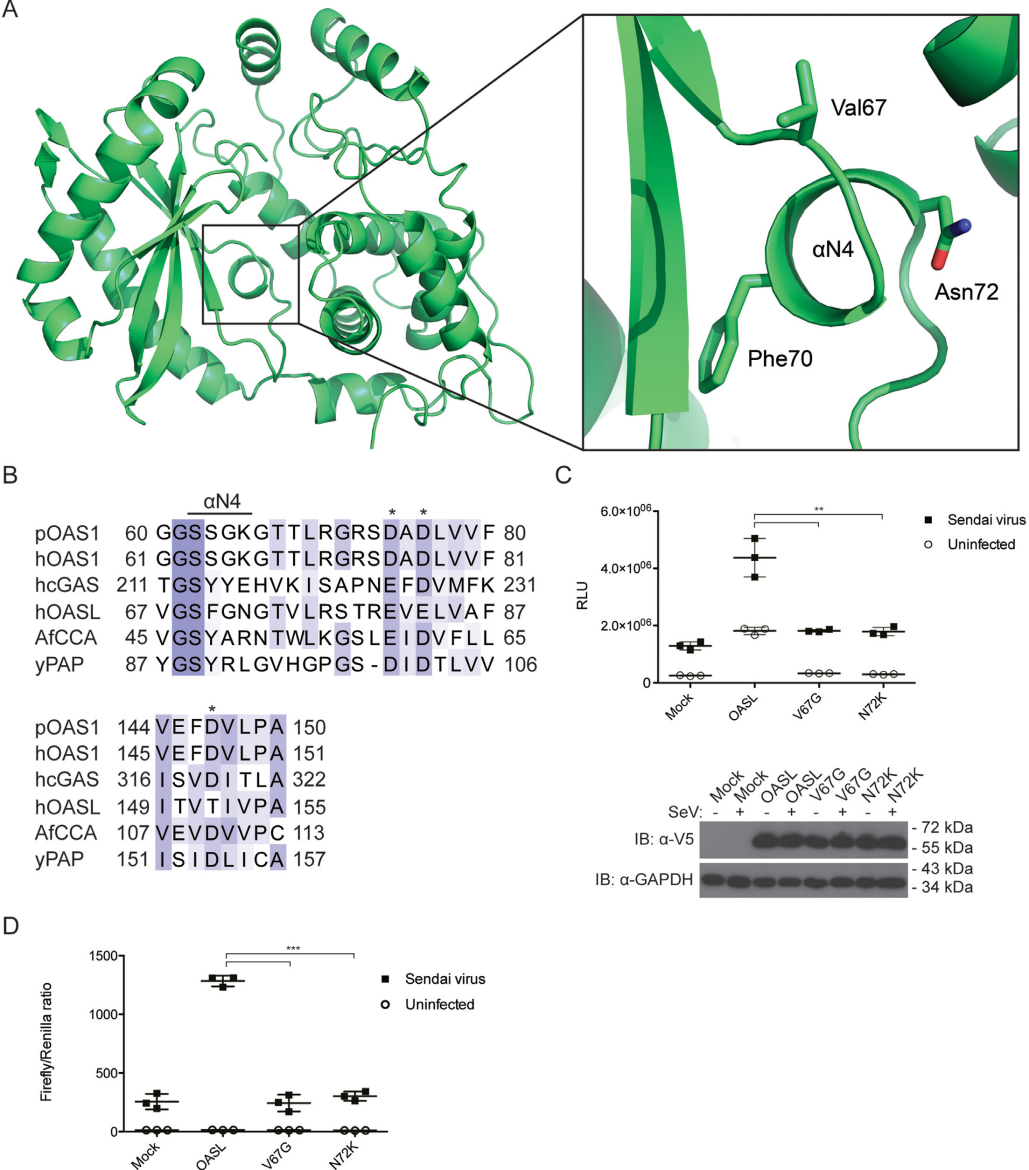
The αN4 is an integral part of OASL. (**A**) The crystal structure of OLD with a zoom-in on the αN4 helix. The anchoring residues Val67, Phe70 and Asn72 are indicated and their side chains are shown in sticks. (**B**) An alignment of porcine OAS1 (pOAS1), human OAS1 (hOAS1), human cGAS (hcGAS), human OASL (hOASL), *Archaeoglobus fulgidus* CCA (AfCCA) and *Saccharomyces cerevisiae* PAP (yPAP). The residues spanning the αN4 or the αN4-equivalent helices in cGAS, CCA and PAP are indicated. The asterisks indicate the three carboxyl acid residues in the catalytic triad of OAS1, cGAS, CCA and PAP. The residues are colored according to conservation. (**C**) HEK293T OASL^neg^ cells were co-transfected with V5-tagged empty pcDNA3.1 vector (mock), OASL, OASL V67G or OASL N72K in combination with an IFN-β promoter firefly luciferase reporter construct for 24 h. The cells were either infected with SeV or mock infected for 16 h and the luciferase activities were measured. Error bars indicate standard deviation from three independent experiments. The inset shows an immunoblot against V5 and GAPDH to confirm the expression of the transfected constructs and to ensure that similar amounts were loaded on the gel, respectively. (**D**) HeLa cells were co-transfected with V5-tagged empty pcDNA3.1 vector (mock), OASL, OASL V67G or OASL N72K in combination with an IFN-β promoter firefly luciferase reporter construct and a β-actin promoter *Renilla* luciferase construct for 24 h. The cells were either infected with SeV or mock infected for 16 h and the luciferase activities were measured. The measurements are shown as a ratio of firefly to *Renilla* luciferase activities. Error bars indicate standard deviation from three independent experiments. **: *P*-value ≤ 0.01. ***: *P*-value ≤ 0.001.

Crystallization conditions for the OLD were established using standard commercial screens and the optimized crystals diffracted to 1.6 Å (Table 1). Phases were determined by molecular replacement using the structure of porcine OAS1 as a search model. An initial model was built automatically and completed by cycles of manual building and refinement (Table 1). Overall, the electron density of the OLD was well defined (Figure 2A). However, no density was observed for the four most N-terminal residues or for the two most C-terminal residues and the C-terminal 6xHis-tag. Likewise, two loop regions spanning the residues 52–56 and 182–186 did not display well-defined electron density. The structure of the OLD (PDB: 4XQ7) adopts a classical OAS structure consisting of two lobes connected by a linker region and an N-terminal extension (Figure 2B) (35,36). The N-terminal lobe harbors the five-stranded β-sheet found in all polymerases. The β-sheet is flanked by the two α-helices αN3 and αN5. The C-terminal lobe is a four-helix bundle with a three-stranded mixed β-sheet with an additional interstrand α-helix connection (Figure 2B). The OLD displays a remarkable similarity with the activated form of OAS1 (PDB: 4IG8) with an RMSD of 1.6 Å and a Z-score of 39.7 calculated using the Dali server. The RMSD and Z-score toward the inactive form of OAS1 (PDB: 1PX5) are 2.4 Å and 35.6, respectively. Notably, only after the binding of dsRNA does OAS1 form a rigid αN4 helix, which participates in coordinating the triphosphate of the donor ATP in 2-5A synthesis, however, in OASL the αN4 is intrinsically present (Figure 2C). In the absence of dsRNA, the residues constituting the αN4 in OAS1 are positioned in a highly flexible loop leading to ill-defined electron density in the crystal structure. However, the dsRNA-induced structural rearrangements lead to the formation of the αN4 from the previously disorganized residues (Figure 2C). Species difference between the two determined OAS1 structures is unlikely to affect the structural differences between the inactive and active OAS1 conformations. The structurally related cGAS undergoes similar rearrangements when activated by dsDNA (this is discussed in (62)). In fact in the apo-form, cGAS also has a disordered region that after dsDNA-induced structural rearrangement becomes ordered and forms an αN4-equivalent helix (27). Additionally, to the αN4 formation, the dsRNA-induced structural rearrangement in OAS1 is mirrored in the position of the αN3 and αN5 helices in the OLD (Figure 2D). For a comprehensive overview of the shared and unique secondary structure features between the OLD and the active and inactive OAS1 conformations see Figure 3. OASL intrinsically forms the αN4 helix due to residues Val67, Phe70 and Asn72 that anchor the loop and form the αN4 helix (Figure 4A). The intrinsic formation of the αN4 in the OLD is reminiscent of the structures of the constitutively active NTases poly(A) polymerase (PAP) and CCA-adding enzyme (CCA) (63,64). The constitutively active PAP and CCA form an αN4-equivalent helix in a similar fashion by bulky and hydrophobic residues, which anchor the helix and ensure constitutive activity (Figure 4B). We used HEK293T cells in which the OASL gene has been knocked out (OASL^neg^) and transiently transfected them with empty vector (mock) or plasmids expressing wild-type OASL or the mutants V67G or N72K and then measured RIG-I signaling in response to Sendai virus (SeV) infection by an IFN-β promoter firefly luciferase reporter construct. Mutating the key residues Val67 and Asn72 in the αN4 helix to the corresponding residues found in OAS1 completely abrogates the enhancement of RIG-I signaling (Figure 4C), indicating that the αN4 plays an important role in OASL function. We noticed that IFN-β signaling was increased in uninfected HEK293T OASL^neg^ cells in a manner that was dependent on a functional OASL. This effect is not due to differences in transfection efficiencies but is likely an artifact of overexpressing OASL, which is commonly observed when overexpressing other IFN-β promoter activating proteins such as RIG-I or stimulator of IFN genes (STING). We repeated the IFN-β reporter assay in HeLa cells co-transfected with a β-actin promoter *Renilla* luciferase reporter construct, which was subsequently used for normalization (Figure 4D). HeLa cells exhibited much lower IFN-β promoter activity in the absence of infection but otherwise the data obtained from the two different cell lines and with and without using *Renilla* luciferase for normalization agree well. The intrinsic formation of the αN4 could potentially indicate that OASL retains NTase activity or nucleotide binding properties. To investigate the NTase activity of OASL, we used the purified OLD and full-length OASL from insect cells as well as IP OASL from stable transfected Flp-In T-Rex HEK293 cells to ensure the presence of possible unknown cofactors. OASL was incubated with all four nucleotides and activating dsRNA or dsDNA but displayed no NTase activity (Supplementary Figure S2).

**Table 1.** Data collection and refinement statistics of OLD

	OLD
**Data collection**	
Wavelength (Å)	0.97907
Space group	P 1 2_1_ 1
Unit cell dimensions	
*a, b, c* (Å)	54.8, 57.62, 64.79
α, β, γ (°)	90, 99.44, 90
Resolution range (Å)	35.51 - 1.6 (1.657 - 1.6)
Total reflections	198209 (19682)
Unique reflections	52563 (5258)
Multiplicity	3.8 (3.7)
Completeness (%)	99.80 (99.92)
*I*/σ*I*	13.97 (1.82)
Wilson B-factor	13.93
*R*_merge_ (%)	7.312 (75.49)
*R*_meas_ (%)	8.534
CC1/2	0.998 (0.636)
CC*	1 (0.882)
**Refinement**	
*R*-work (%)	16.71
*R*-free (%)	19.26
Number of non-hydrogen atoms	3151
Protein	2749
Ligands	0
Water	402
Protein residues	334
Rms deviations	
Bond length (Å)	0.01
Bond angles (°)	1.2
Ramachandran	
Favored (%)	98
Allowed (%)	2
Outliers (%)	0
Clashscore	3.83
Average B-factor	21.50
Protein	19.4
Solvent	30.5

Values in the parenthesis are the values for the highest resolution shells.

### OASL requires dsRNA binding to enhance RIG-I signaling

**Figure 5.**
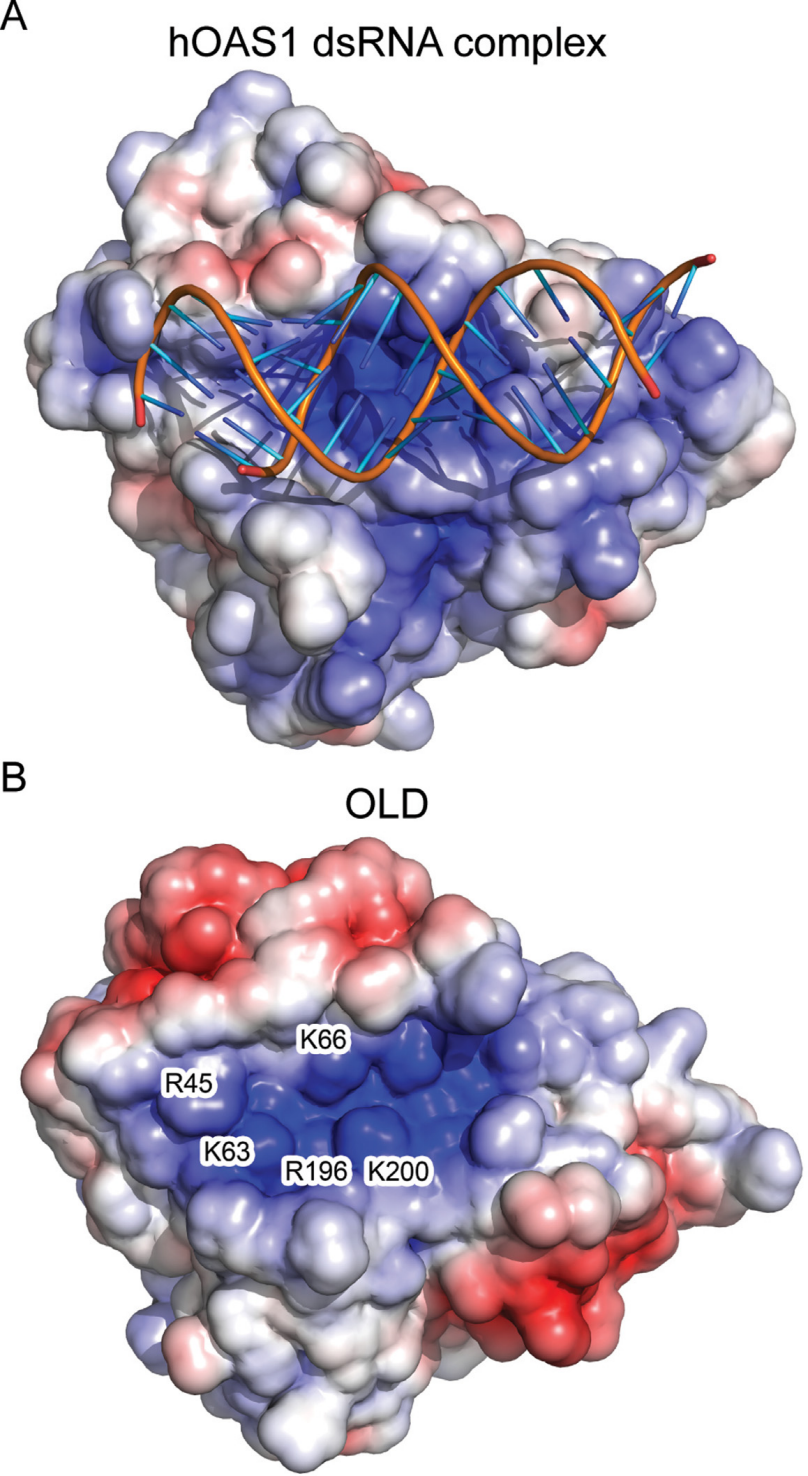
OASL has a dsRNA binding groove. (**A**) Surface charge representation of human OAS1 in complex with dsRNA (PDB: 4IG8). (**B**) Surface charge representation of the OLD (PDB: 4XQ7). Residues in the dsRNA binding groove that are mutated in Figure 6E are indicated at their position. The surface charge colors range from +5 to -5 k_b_T/e_c_.

**Figure 6.**
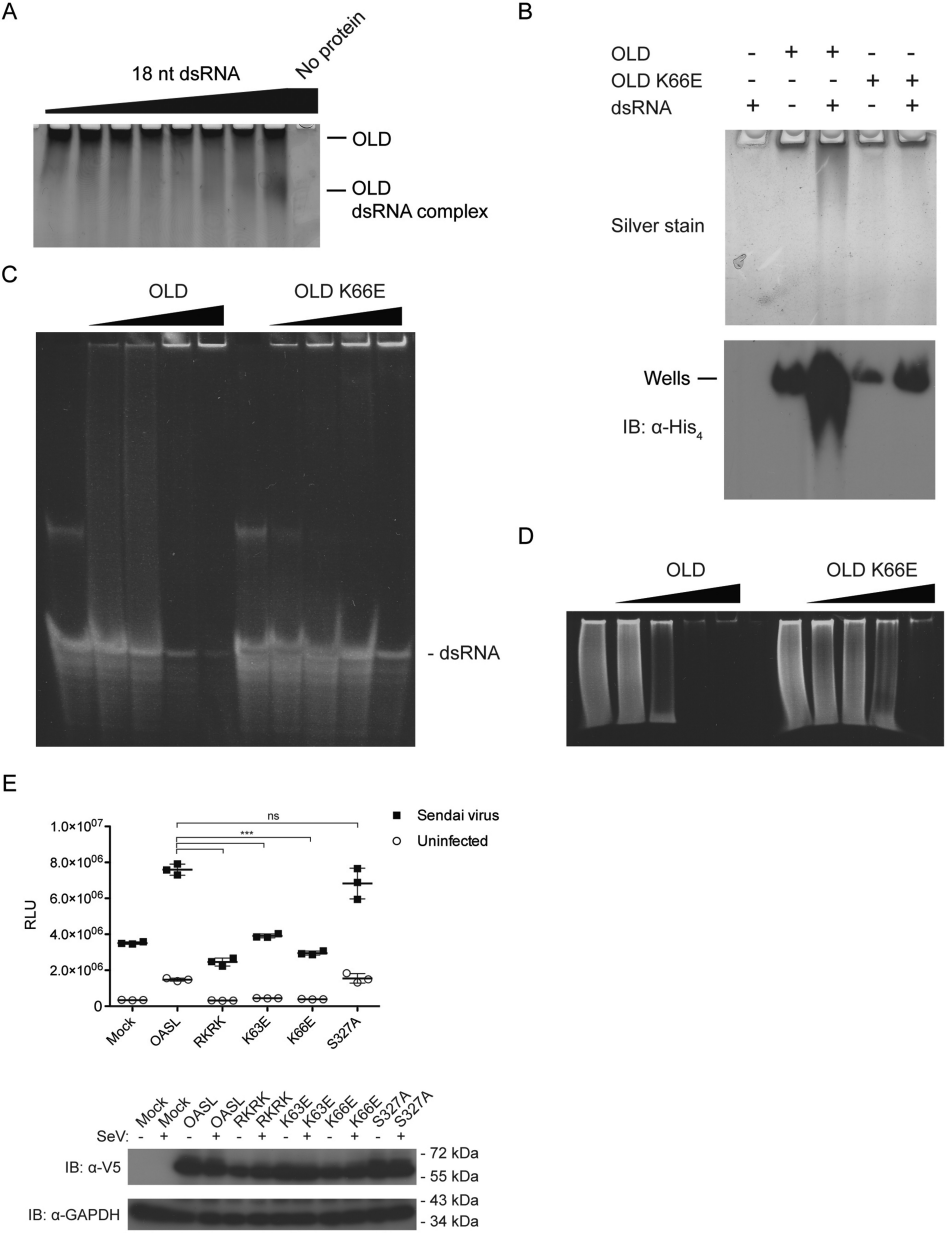
OASL binds to dsRNA and dsRNA binding is essential to enhance RIG-I signaling. (**A**) A constant concentration of OLD was incubated with increasing amounts of 18 bp dsRNA and subsequently analyzed by silver stained native PAGE. OLD was kept constant at 2.4 μM (1 μg) and incubated with 0, 0.13, 0.27, 0.54, 1.1, 2.2, 4.3 or 8.6 μM (1 μg) 18 bp dsRNA. The negative control was performed with 8.6 μM 18 bp dsRNA and no OLD. (**B**) 2.4 μM wild-type OLD or K66E OLD was incubated with 8.6 μM 18 bp dsRNA and analyzed by native PAGE. The proteins were visualized by silver staining or immunoblotting against the 6xHis-tag. Wells: the position of the bottom of the wells in the corresponding native gel. (**C**) A constant concentration of 18 bp dsRNA was incubated with increasing amounts of either wild-type OLD or the K66E mutant and subsequently analyzed by native PAGE. The concentration of 18 bp dsRNA was kept constant at 10 μM and incubated with 0, 7, 28 or 56 μM OLD wt or K66E. The nucleic acid was visualized by ethidium bromide staining. (**D**) A constant concentration of poly(I:C)-LMW was incubated with increasing amounts of either wild-type OLD or the K66E mutant and subsequently analyzed by native PAGE. The concentration of poly(I:C)-LMW was kept constant at 0.2 mg/ml and incubated with 0, 7, 28 or 56 μM OLD wt or K66E. The nucleic acid was visualized by ethidium bromide staining. (**E**) HEK293T OASL^neg^ cells were co-transfected with V5-tagged empty pcDNA3.1 vector (mock), OASL, OASL R45E/K66E/R196E/K200E (RKRK), OASL K63E, OASL K66E or OASL S327A in combination with an IFN-β promoter luciferase reporter construct for 24 h. The cells were either infected with SeV or mock infected for 16 h and the luciferase activities were measured. Error bars indicate standard deviation from three independent experiments. The inset shows an immunoblot against V5 and GAPDH to confirm the expression of the transfected constructs and to ensure that similar amounts were loaded on the gel, respectively. Ns: *P*-value > 0.05. ***: *P*-value ≤ 0.001.

OAS1 utilizes a positively charged groove to bind dsRNA (Figure 5A) (36). A surface charge representation of the OLD shows a putative dsRNA binding groove in OASL (Figure 5B). To investigate the ability of the OLD to bind to dsRNA *in vitro*, we incubated it with increasing amounts of 18 bp dsRNA and performed EMSAs using native PAGE. The OLD alone migrated slowly into the gel due to its small negative charge, however, in the presence of increasing amounts of dsRNA, OLD more readily migrated into the gel due to the formation of a complex with a larger net negative charge (Figure 6A). To determine whether the OLD binds dsRNA specifically through the putative dsRNA binding groove, we introduced a charge-swap mutation, K66E, within this groove and then repeated the EMSA with wild type and the K66E mutant forms of OLD. Compared to the wild-type OLD, the K66E mutant showed a much weaker electrophoretic shift in the presence of dsRNA (Figure 6B). Vice versa, when we incubated a fixed amount of dsRNA with increasing amounts of the OLD, the dsRNA was increasingly retained leading to weaker bands of dsRNA (Figure 6C). The K66E mutant was impaired in retaining dsRNA although the impairment could be overcome to some degree by a higher concentration of K66E compared to wild type (Figure 6C). In a similar fashion, the OLD also retained low molecular weight poly(I:C) (a dsRNA analog with a size from 0.2 to 1 kb) whereas the K66E mutant is impaired in poly(I:C) retention (Figure 6D). This indicates that OASL binds dsRNA and that the K66E mutant has a reduced affinity toward dsRNA. To investigate the functional relevance of dsRNA binding, we introduced charge-swap mutations in the putative dsRNA binding groove of full-length OASL. The four residue mutant R45E/K66E/R196E/K200E (RKRK) and the single residue mutants K63E and K66E were all incapable of enhancing RIG-I signaling, whereas mutating the unrelated surface exposed Ser327 to Ala had no effect (Figure 6E). These results show that OASL binds dsRNA through the positively charged groove and that dsRNA binding is important for OASL to enhance RIG-I signaling.

## DISCUSSION

OASL is a protein that was recently shown to belong to the RIG-I enhanceosome (44). Initial virus detection induces the expression of OASL directly via IRF3 or as an IFN-stimulated gene (20). Upon subsequent virus detection, OASL can promote RIG-I signaling thereby enhancing the antiviral response during secondary virus recognition. As mentioned, the UbLD has homology to two ubiquitin domains and like K63-linked ubiquitin it has been shown to bind the CARDs of RIG-I (44). RNA and pUb/UbLD binding to RIG-I is part of a two-step activation mechanism that renders RIG-I capable of interacting with MAVS (8). In agreement with this model, truncating OASL so it lacks the UbLD abolishes its antiviral activity (44,45). The OLD is suggested to interact either directly or indirectly with the CTD of RIG-I (44), however, the functional significance of the OLD has remained unknown.

The crystal structure of the OLD exhibits a high similarity with active OAS1 and both proteins form the αN4 helix, which is absent in inactive OAS1 (Figure 2) (35,36). The OAS proteins as well as cGAS, PAP and CCA belong to a structurally but not functionally related NTase superfamily (65). The constitutively active NTases, e.g. PAP and CCA intrinsically form equivalents of the αN4 helix found in the active form of OAS1 (36,63–64). The αN4 helix or equivalent helices is a common motif that facilitates nucleotide binding and this helix is also known as the phosphate-binding loop (P-loop) (66,67). The binding of double-stranded nucleic acids to OAS1 and cGAS induce the formation of the P-loop, which in part explains their requirement for nucleic acid binding for NTase activity. For example, in the activated OAS1, the Ser63 residue (Figure 4B) coordinates the triphosphates of the donor ATP during 2-5A synthesis (36). The constitutively active PAP and CCA utilizes bulky and hydrophobic residues to anchor the P-loop for catalysis (Figure 4B). The OAS enzymes likely originate from constitutively active NTases such as PAP and CCA but have evolved into dsRNA-activated enzymes with unanchored P-loops and thus without the intrinsic formation of the αN4 (68). Like many other mammals, mice harbor two *Oasl* genes. In contrast to human OASL and murine OASL1, murine OASL2 is enzymatically active and requires dsRNA for activity reflecting the fact that OASL must have arisen from an ancestral catalytically active OAS1-like gene (69). *OASL2* can also be found in humans but only as a pseudogene. The intrinsic formation of αN4 in the constitutively active NTases is a feature that was most likely lost once the OAS enzymes evolved into dsRNA-activated enzymes. However, this feature was apparently reacquired in human OASL (and likely also its orthologs) later in evolution. A key residue for the intrinsic αN4 formation in human OASL is the Phe70 (Figure 4A). The equivalent residue to the human OASL Phe70 is always a Ser in active OAS isoforms but either a Phe or Tyr in PAP and interestingly this residue is also a Tyr in cGAS (Figure 4B) (31). Although OASL is catalytically inactive in part due to its incomplete catalytic triad (Supplementary Figure S2), perturbation of the αN4 helix by mutagenesis is detrimental to the function of OASL suggesting that the proper formation of the αN4 in OASL is required (Figure 4C). As formation of the αN4 in OAS1 is connected to dsRNA binding, ‘loosening’ the αN4 in OASL by mutating key residues to the corresponding residues in OAS1 potentially abrogates the conformation of the dsRNA binding site. This could lower the affinity for dsRNA, which might explain the phenotype observed for the V67G and N72K mutants (Figure 4C and D).

Surface charge representations of the OLD demonstrate a positively charged groove that could potentially facilitate dsRNA binding in a similar fashion as for OAS1 (Figure 5). Using an 18 bp dsRNA which binds to human OAS1 (36), as well as the dsRNA analog poly(I:C), we demonstrate that dsRNA binding is indeed a feature of the OLD. Mutating the key residues within the positively charged groove not only reduces the affinity toward dsRNA but also abrogates the ability of OASL to enhance RIG-I signaling (Figure 6). Hence, binding to dsRNA plays a central role in the function of OASL. The recent finding that dsRNA binding to a catalytically inactive OAS domain has functional relevance in OAS3 corroborates our results (70). Taken together these studies indicate that catalytically inactive OAS domains can play important functional roles as putative dsRNA sensors. The role of dsRNA binding to OASL in regard to RIG-I signaling is currently unknown. However, activation of RIG-I is tightly regulated by several mechanisms in order to prevent undue activation of the innate immune response. In contrast to K63-linked pUb, our data suggests that OASL has to bind dsRNA before it can enhance RIG-I signaling, which could present a fail-safe mechanism meant to prevent aberrant activation of RIG-I. Alternatively, OASL could modify the specificity of RIG-I toward dsRNA possibly by shared RNA binding and thereby allow RIG-I to detect a wider range of RNAs when in complex with OASL. Shared RNA binding between OLD and RIG-I could also position UbLD and CARD in close proximity and thereby facilitate the interaction.

In conclusion, our data show that the enhancement of RIG-I signaling requires the coordinated action of both domains of OASL, demonstrating essential functional roles of both the OLD and UbLD in RIG-I signaling enhancement.

## SUPPLEMENTARY DATA

Supplementary Data are available at NAR Online.

SUPPLEMENTARY DATA
