# Supplementary material for: Structural and functional analysis reveals that human OASL binds dsRNA to enhance RIG-I signaling
Source: Nucleic Acids Res. 2015 Apr 29;43(10):5236–48. doi: 10.1093/nar/gkv389 (PMC4446440; doi:10.1093/nar/gkv389)
Supplement: SUPPLEMENTARY DATA [file supp_43_10_5236__index.html]

Structural and functional analysis reveals that human OASL binds dsRNA to enhance RIG-I signaling — Structural and functional analysis reveals that human OASL binds dsRNA to enhance RIG-I signaling — SUPPLEMENTARY DATA 

# Structural and functional analysis reveals that human OASL binds dsRNA to enhance RIG-I signaling

## SUPPLEMENTARY DATA

**Files in this Data Supplement:**

- SUPPLEMENTARY DATA
- SUPPLEMENTARY DATA
