# Supplementary material for: Structural and functional analysis reveals that human OASL binds dsRNA to enhance RIG-I signaling
Source: Nucleic Acids Res. 2015 Apr 29;43(10):5236–48. doi: 10.1093/nar/gkv389 (PMC4446440; doi:10.1093/nar/gkv389)
Supplement: SUPPLEMENTARY DATA [file supp_gkv389_nar-00142-r-2015-File009.pdf]

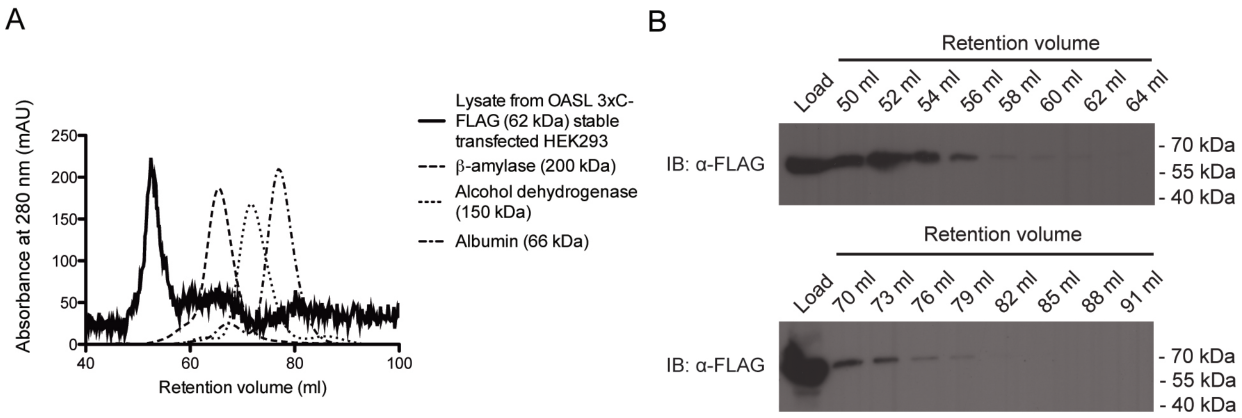


Supplementary figure 1. Full-length OASL forms soluble multimers when expressed in human cells. (A) Chromatograms of Superdex 200 HiLoad 16/60 size exclusion chromatography of OASL lysate from stable transfected Flp-In T-Rex HEK293, β-amylase, alcohol dehydrogenase and albumin in solid, dashed, dotted and interchanging lines, respectively. (B) Anti-FLAG immunoblot of the lysate loaded onto the column (load) and retention volumes from the size exclusion chromatography shown in (A).

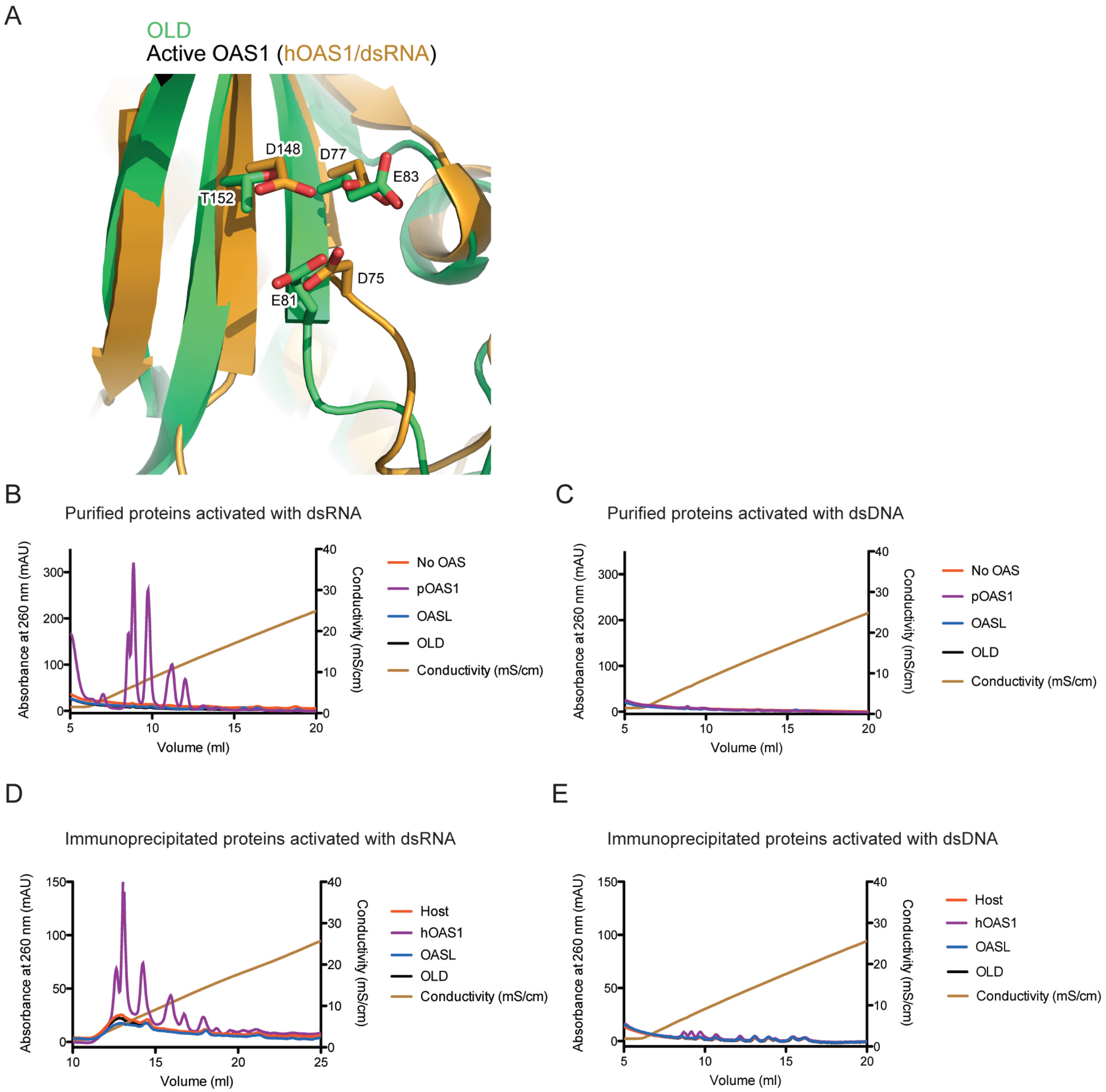


Supplemental figure 2. OASL does not harbor catalytic activity involving nucleotides. (A) Superposition of OLD (green, PDB: 4XQ7) on active OAS1 (human OAS1 in complex with dsRNA, yellow, PDB: 4IG8). The side chains of the three catalytic aspartic acid residues in OAS1 Asp75, Asp77 and Asp148 are shown and indicated. The side chains of the corresponding residues in OASL Glu81, Glu83 and Thr152 are shown and indicated. (B) Purified pOAS1, OASL and OLD were assayed for activation by the dsRNA analog poly(I:C) and synthesized products were bound to a 1 ml Resource Q column. Bound products were eluted from the column in a linear gradient of NaCl. Absorption peaks correspond to the eluted 2-5As synthesized by pOAS1. (C) Similar to (B), however, pOAS1, OASL and OLD were activated with salmon sperm dsDNA. (D) Similar to (B) but immobilized immunoprecipitated proteins from Flp-In T-Rex HEK293 cells stable transfected with hOAS1, OASL, OLD or non-transfected cells (Host) were assayed for activity. All proteins have 3xC-terminal FLAG-tags for immunoprecipitation. The immunoprecipitated proteins immobilized on beads were used and activated by dsRNA. (E) Similar to (D) but activated with dsDNA.
